# The feasibility of a novel injectable hydrogel for protecting artificial gastrointestinal ulcers after endoscopic resection: an animal pilot study

**DOI:** 10.1038/s41598-021-97988-9

**Published:** 2021-09-16

**Authors:** Yuko Miura, Yosuke Tsuji, Rina Cho, Ayano Fujisawa, Masahiko Fujisawa, Hiroyuki Kamata, Yuki Yoshikawa, Nobutake Yamamichi, Takamasa Sakai, Kazuhiko Koike

**Affiliations:** 1 Department of Gastroenterology, Graduate School of Medicine, The University of Tokyo, 7-3-1, Hongo, Bunkyo-ku, Tokyo, 113-8655 Japan; 2 Department of Bioengineering School of Engineering, The University of Tokyo, Tokyo, Japan; 3 Department of Basic Science, School of Veterinary Nursing and Technology, Faculty of Veterinary Science, Nippon Veterinary and Life Science University, Tokyo, Japan

**Keywords:** Gastroenterology, Materials science

## Abstract

Recently, covering materials for protecting post-endoscopic ulcers are being developed using hydrogels. Existing hydrogels are not ideal coating materials because it is difficult to control their physical properties. Therefore, we conducted an animal pilot study to investigate the protective effect of a novel ulcer coating material, whose physical properties can be easily controlled and designed. We applied the novel injectable hydrogel to artificial ulcers induced on the gastric mucosa of rats. Rats were assigned to the hydrogel or the control group. To measure the protective effect of hydrogel on ulcers, the perforation rate, ulcer diameter, and ulcer area were evaluated 48 h after gel application. As secondary endpoints, we assessed the residual rate of the hydrogel at the bottom of the ulcer, performed histological analysis, and analyzed adverse events associated with hydrogel. The perforation rate was significantly lower (16% vs. 75%) and the mean diameter of ulcers was significantly smaller (5.4 ± 1.8 mm vs. 7.8 ± 2.8 mm) in the hydrogel group. Histopathological findings revealed the inflammatory cell count was significantly higher in the control group. Our novel hydrogel showed a protective effect on artificial gastric ulcers in a rat model.

## Introduction

Endoscopic submucosal dissection (ESD) is the standard treatment method for superficial neoplasms in the gastrointestinal tract in many countries. Although ESD is a minimally invasive treatment compared to surgery^1,2^, adverse events frequently occur. Major adverse events associated with ESD include post-ESD bleeding and delayed perforation^3,4^. Post-ESD bleeding may lead to serious hemorrhagic shock requiring blood transfusion or urgent surgery^5,6^. In most cases, delayed perforation, especially after colorectal ESD, requires emergency surgery^7^. Therefore, prevention of these incidents is clinically important.

Post-polypectomy coagulation syndrome (PPCS) is known to have symptoms similar to those of perforation^8,9^. PPCS refers to the onset of symptoms such as abdominal pain, fever, increased leukocytosis, and peritonitis after endoscopic treatment, without perforation. PPCS occurs as a result of electrocoagulation injury to the bowel wall that induces a transmural burn and localized peritonitis, resulting in serosal inflammation. With the evolution of treatment, the occurrence of PPCS is increasing, with an incidence of 7–8% among patients after ESD^9^. Although most cases of PPCS have an excellent prognosis, PPCS can cause delayed perforation. PPCS ranges in severity, and can lead to shock and additional surgery^8^. Therefore, these adverse events should be prevented whenever possible.

Several reports have analyzed the efficacy of protecting wounds after ESD^8–10^. Wound suturing with endoclips is frequently used as a first-line treatment. However, ESD-induced ulcers tend to be large (> 20 mm). Previous reports have indicated that complete closure with a suturing technique is difficult to achieve in larger ulcers^11,12^. Hence, wound-shielding techniques have attracted considerable attention. Endoscopic shielding with polyglycolic acid sheets and fibrin glue has been reported to be effective in preventing perforation and bleeding^13–15^. This method is advantageous because it has already been used in many medical fields^16,17^. However, it is technically difficult to accurately attach a sheet-like material to the bottom of the ulcer using an endoscope; therefore, generalized use of this method is difficult.

Recent attempts have been made to develop a covering material for protecting post-endoscopic ulcers using hydrogels^18–21^. Injectable hydrogels, only recently employed in clinical practice, can be easily applied in the liquid form under endoscopic use. However, the existing injectable hydrogels are not ideal coating materials because it is difficult to control their physical properties, such as gelation time and elasticity. These limitations hinder ideal gelation from occurring at the bottom of the ulcer; thus, adequate coverage of the ulcer base may not be achieved.

Sakai et al. have developed a novel hydrogel, Tetra-PEG gel, with a highly uniform network structure. Tetra-PEG gel is formed from tetra-functional polyethylene glycol precursors; as its physical properties can be freely designed, it is easy to control^22^. In this study, we focused on Tetra-PEG gel because its highly designable features make it possible to create an ideal covering material. We conducted an animal pilot study to investigate the protective effect of a novel ulcer coating material using Tetra-PEG gel.

## Results

**Figure 1:**
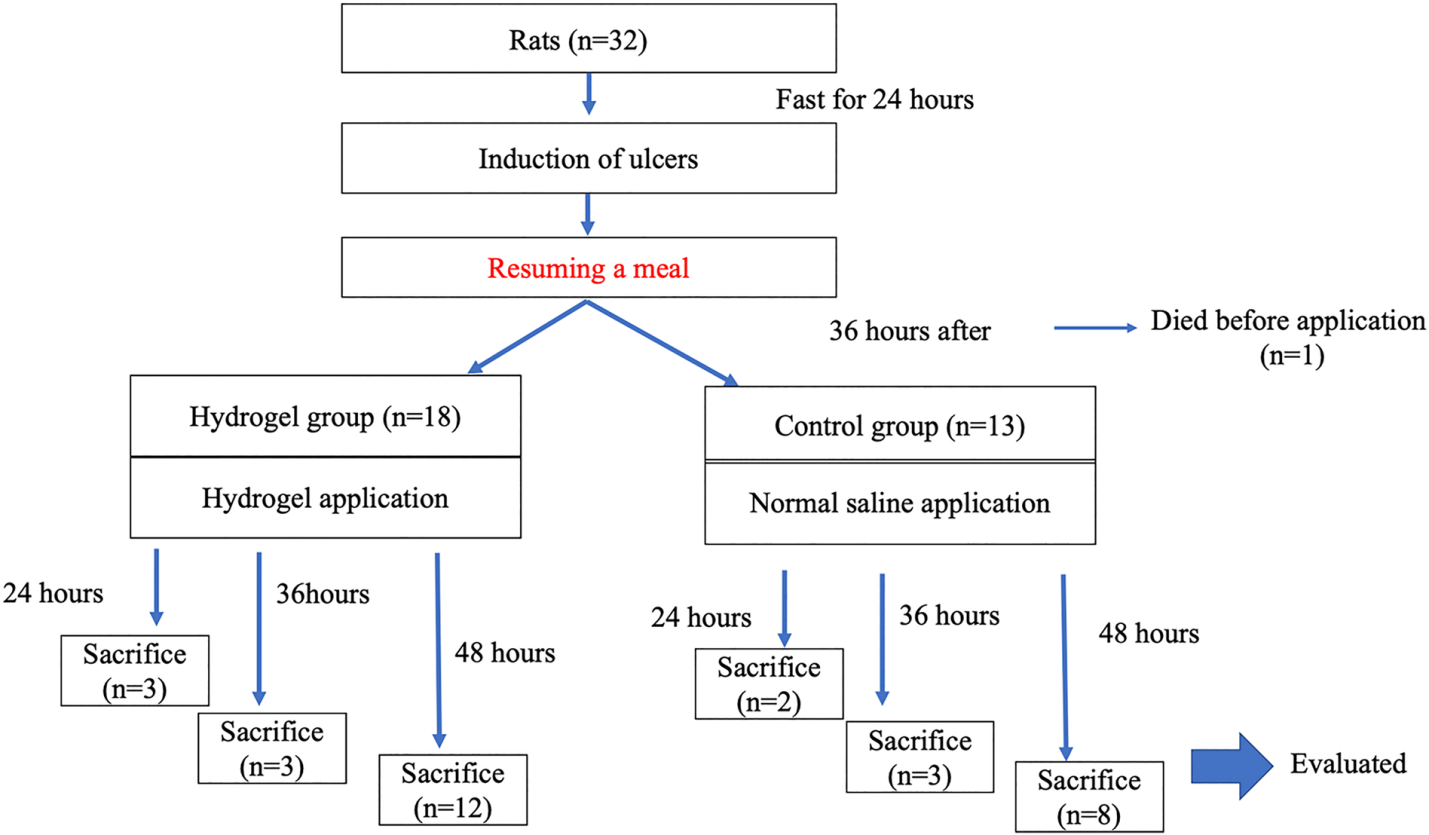
Flowchart of the present study.

Thirty-two rats were used in this study. After excluding one rat that died before gel application, 31 rats were included in the analysis: 24 h, three rats in the hydrogel group and two in the control group; 36 h, three rats in each group; and 48 h, 12 rats in the hydrogel group and eight in the control group. A flowchart of the study protocol is presented in Fig. 1.

The perforation rate at 48 h was significantly lower in the hydrogel group than in the control group (16% vs. 75%; *P* = 0.02). The mean diameter of ulcers 48 h after hydrogel application was significantly smaller in the hydrogel group than in the control group (5.4 ± 1.8 mm vs. 7.8 ± 2.8 mm; *P* = 0.03). The mean ulcer area after 48 h tended to be smaller in the hydrogel group, but this difference did not reach statistical significance (21.0 ± 14.4 mm^2^ vs. 33.5 ± 21.3 mm^2^; *P* = 0.13). The percentage of hydrogel remaining at the bottom of the ulcer was 66% after 24 h, 33% after 36 h, and 16% after 48 h. Adverse events associated with gel application were not observed (Table 1).

**Table 1:** Macroscopic findings 48 h after hydrogel or saline application.

	Hydrogel group (n = 12)	Control group (n = 8)	*P* value
Rate of perforation, n (%)	2 (16)	6 (75)	0.02
Mean ulcerated diameter, mm (mean ± SD)	5.4 ± 1.7	7.8 ± 2.8	0.03
Mean ulcerated area, mm^2^ (mean ± SD)	21.0 ± 13.8	33.5 ± 19.9	0.13
Hydrogel remaining on bottom of ulcer after 48 h, n (%)	2 (16)	–	–
Adverse events (%)	0	0	–

*SD* Standard deviation.

**Figure 2:**
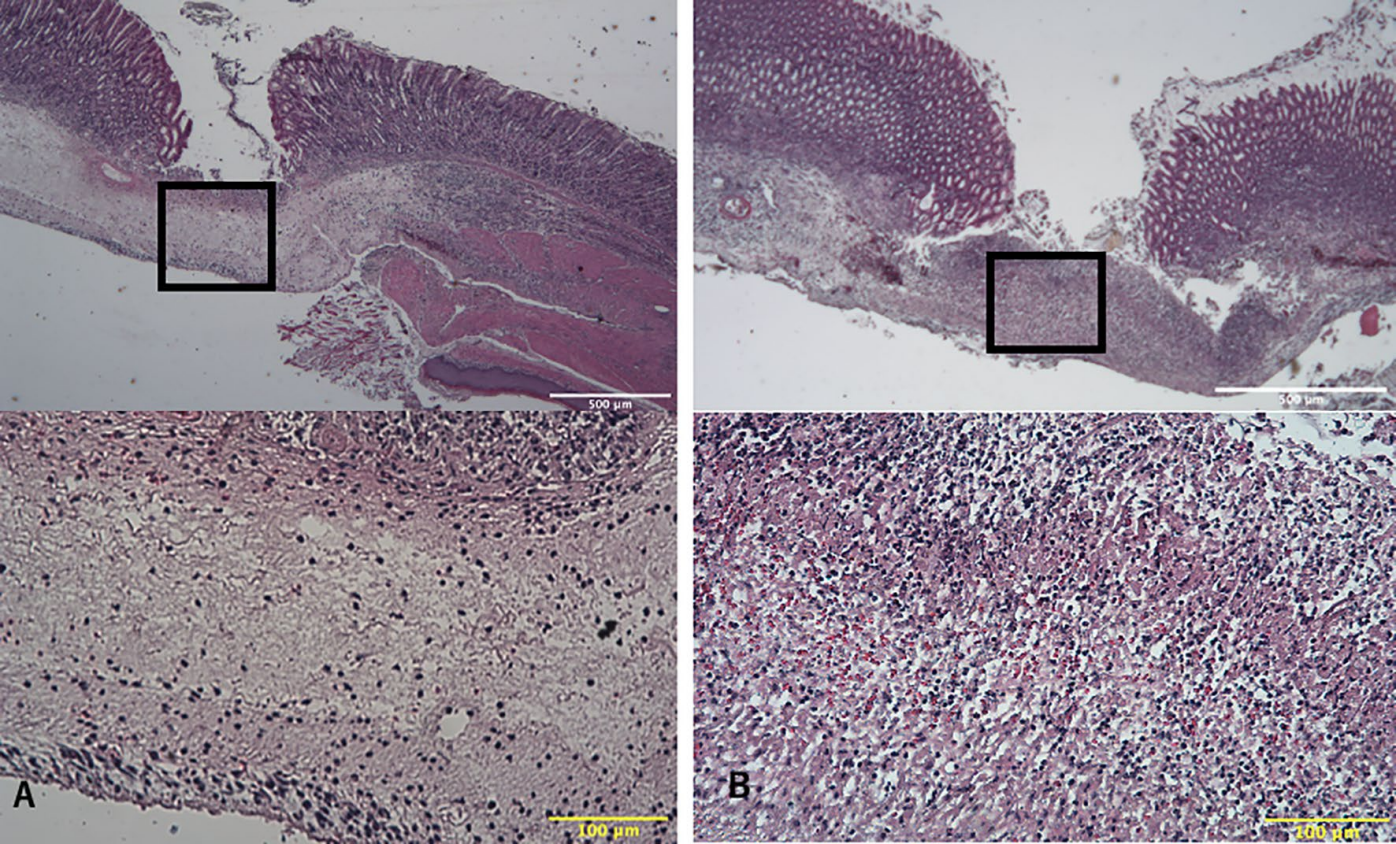
Histopathology findings. (**A**) Microscopic view of the ulcer 48 h after hydrogel application in the hydrogel group. (**B**) Microscopic view of the ulcer 48 h after saline application in the control group. Black squares indicate the mucosal defect area. The density of the number of inflammatory cells in the mucosal defect area was assessed. Yellow bars indicate 100 μm.

**Figure 3:**
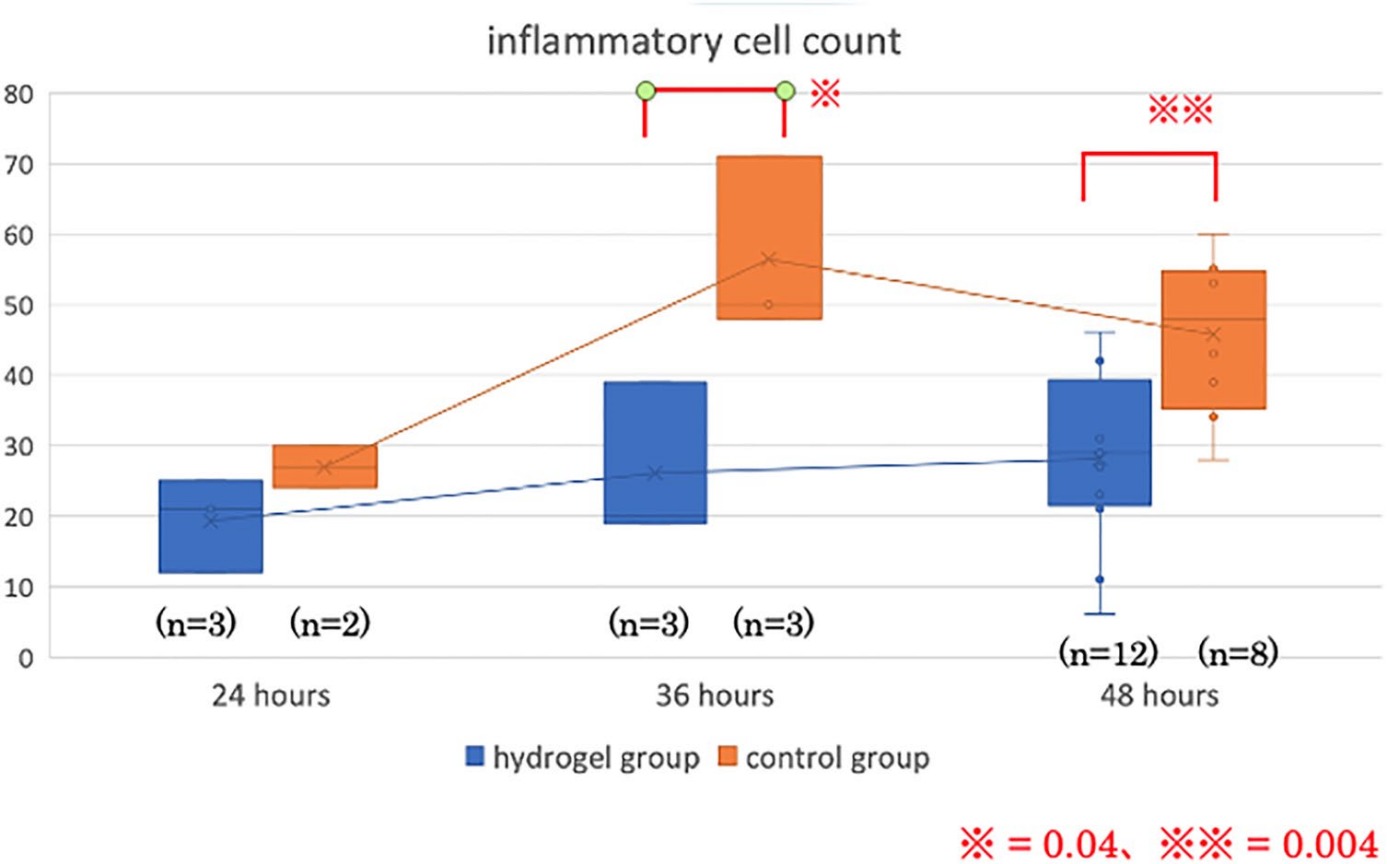
The number of inflammatory cells per 100 μm^2^ in the deep submucosal layer in the mucosal defect area in the hydrogel and control groups.

Histopathological findings 48 h after gel application are shown in Fig. 2. Inflammatory cells were densely infiltrated in the control group. For quantitative evaluation, the number of inflammatory cells per 100 μm^2^ in the deep submucosal layer in the mucosal defect area was counted. The number of inflammatory cells was significantly higher in the control group than the hydrogel group at both 36 h (56 ± 13 cells/10,000 μm^2^ vs. 26 ± 11 cells/10,000 μm^2^; *P* = 0.04) and 48 h after gel application (46 ± 11 cells/10,000 μm^2^ vs. 28 ± 12 cells/10,000 μm^2^; *P* = 0.004) (Fig. 3).

## Discussion

In the present study, we demonstrated the protective effect of a novel injectable hydrogel on artificial gastric ulcers induced by acetic acid in a rat model. Shielding methods after endoscopic treatment have begun to attract attention, with hydrogels at the forefront of the discussion^18–21^. Previous reports have discussed the preventive effect of hydrogels on intraoperative and postoperative bleeding; however, to the best of our knowledge, no report has found a significantly reduced bleeding rate compared with existing hemostatic procedures. This may be because the hydrogel flows off the bottom of the ulcer before robust gelation. It has also been reported that the position of the ulcer can affect appropriate gelation^20^. These factors could be relevant because the previously reported hydrogels were composed of a single agent, which makes controlling their physical properties difficult.

Previous reports have described clinical experiences using PuraStat® (3-D Matrix Europe, Caluire et Cuire, France), a novel hydrogel composed of a synthetic hemostatic material and administered in a prefilled syringe. Soons et al. showed in a single-arm study that prophylactic PuraStat application after endoscopic mucosal resection is safe and can be performed within a few minutes; however, the postoperative bleeding rate was not different from that reported with existing methods^20^. In a randomized controlled trial, Subramaniam et al. showed that PuraStat is an effective hemostat that can reduce the need for heat therapy to treat bleeding during ESD. However, as in the report by Soons et al., the postoperative bleeding rate was not significantly different between the PuraStat group and the control group^21^.

The hydrogel used in this study has the advantage of controllable properties. The present study showed protective effects in preventing ulcer perforation, similar to the report by Lorenzo et al.^23^ However, notably, Lorenzo et al. used a model of ulcer induced by thermal ablation in the colon, whereas we used a gastric ulcer model to prevent perforation prevention in a gastric acid environment.

Histological findings showed a decrease in the number of inflammatory cells in the hydrogel group compared to that in the control group. This suggests that the Tetra-PEG gel may protect the ulcer by suppressing inflammation and protecting from external mechanical stimuli such as food.

Delayed perforation after endoscopic treatment reportedly occurs within 48 h. In the present study, we evaluated the ulcer status 48 h after application. The percentage of hydrogel remaining at 48 h after application was 16%, which was deemed inadequate. Because this pilot study was conducted in small animals, there is sufficient room to accurately improve the gel application. The problem was attributed to the following factors: limitation of the tool, using a single lumen applicator, blind application, and waiting to match the application timing with gelation. When Tetra-PEG gel is applied with an endoscope, it can be accurately sprayed onto the ulcer base under ideal conditions. This will provide high residual efficacy and protection if the gelation time and elastic modulus can be optimally adjusted. Based on the promising results of the present pilot study, we plan to proceed to a confirmation study using live pigs that can tolerate the endoscope; at that time, we will be able to accurately apply the Tetra-PEG gel to the bottom of the ulcer. In the present study, we set the gelation time at 3–4 min, including the preparation time. Taking advantage of the features of the Tetra-PEG gel allows the gelation to be set instantaneously, and the hydrogel can be sprayed using a double-lumen applicator (Video Clip 1). When Tetra-PEG gel is applied to live pig models, we will be able to spray the gel under direct vision, and the instantaneous gelation setting will provide effective gelation at the ulcer base. Under these conditions, the Tetra-PEG gel is expected to adhere to the ulcer base more firmly. We have already sprayed Tetra-PEG gel on the gastric mucosa of the pig and confirmed its adhesiveness (Video Clip 2).

In conclusion, in this pilot study, we revealed that Tetra-PEG gel might have a protective effect on artificial ulcers. Our future goals are determining the optimal physical properties of Tetra-PEG gel for this purpose and demonstrating that Tetra-PEG gel can exert a protective effect on ulcers in an endoscopic experiment.

## Methods

This study was conducted in accordance with the Guidelines for the Care and Use of Laboratory Animals of the Graduate School of Engineering, University of Tokyo. The study protocol was approved by the Committee for Animal Care at the Graduate School of Engineering, the University of Tokyo (Approval number: KA 17-3, KA20-4). And our study was carried out in compliance with the ARRIVE guidelines (http://www.nc3rs.org.uk/page.asp?id=1357).

A flowchart of the study protocol is presented in Fig. 1. In the present study, we applied Tetra-PEG gel to artificial ulcers induced on the gastric mucosa of rats to investigate the protective effect of Tetra-PEG gel.

### Animals

Six- to eight-week-old male Sprague Dawley rats were used for this study (Charles River Laboratories Japan, Inc., Yokohama, Japan). The rats were habituated under controlled light (12 h light, 12 h dark) and given free access to food and water for at least one week before experimentation. The rats were assigned to either the hydrogel group (n = 18) or the control group (n = 13). Normal saline was administered instead of hydrogel in the control group. In addition, 20 rats were used as a preliminary experiment; therefore, a total of 52 rats were used in our study.

### Ulcer induction

**Figure 4:**
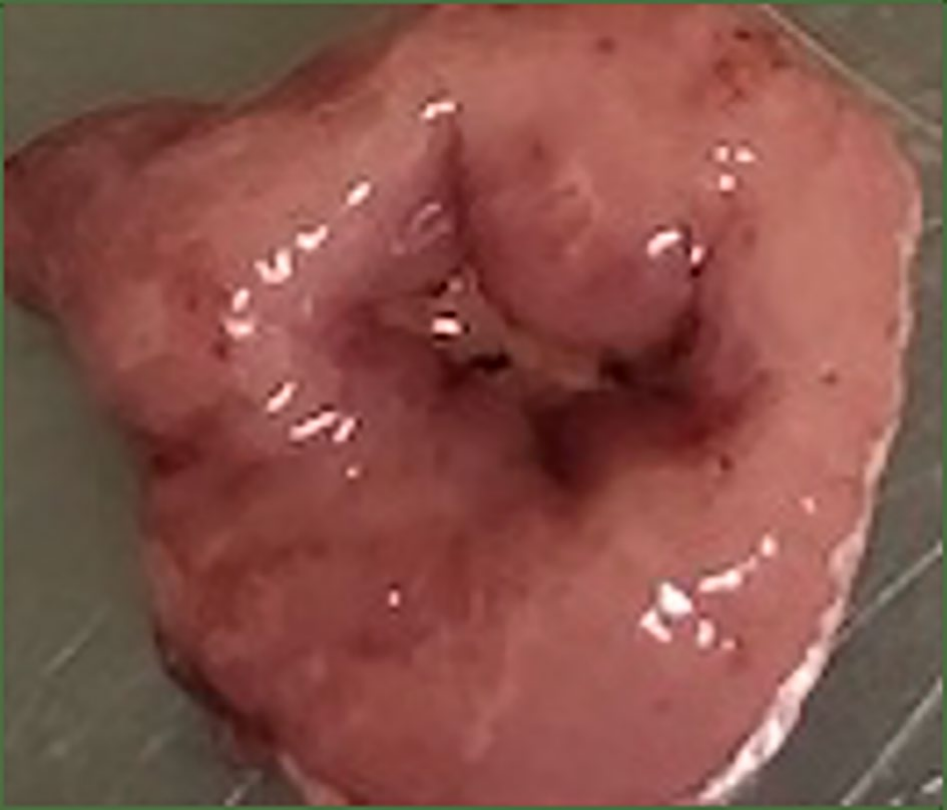
Thirty-six hours after ulcer induction. The ulcer was induced by acetic acid application at 60% v/v concentration for 60 s to an area 8 mm in diameter of the serosal side of the rat stomach.

After a 24-h fasting period, the rats were subcutaneously injected with a mixed anesthetic of medetomidine (0.75 mg/kg), midazolam (4 mg/kg), and butorphanol (5 mg/kg). Under laparotomy, an artificial ulcer was induced just below the esophagogastric junction of each rat based on serosal membrane acetate application ulcers, as described by Okabe et al.^24^ Preliminary experiments revealed that application of acetic acid at a concentration of 60% v/v for 60 s to an area 8 mm in diameter on the serosal side of the rat stomach successfully induced an ulcer of sufficient size and depth (Fig. 4).

Preliminary experiments were conducted to determine the optimal timing for hydrogel application using the ulcer induction method. After experimenting with 20 rats, we obtained the following findings: 1) tissue injury extended from the serosal side to the mucosal side, 2) the ulcer floor became obvious after 6 h, 3) the adhesion of the greater omentum and spleen occurred after 24 h, and 4) the ulcer area was extremely thin and seemed close to perforation 36 h after induction. The mechanism of acetate application ulcers has been reported to be ischemic change due to thrombus formation^24^. Our preliminary experiments showed that the ulceration start from the mucosal side to the serosal side, which was consistent with the previous findings.

### Fabrication and application of Tetra-PEG gels

Tetra-armed sulfhydryl-terminated polyethylene glycol (Tetra-PEG-SH; *M*_w_ = 20 kg/mol) and tetra-armed maleimide-terminated polyethylene glycol (Tetra-PEG-MA; *M*_w_ = 20 kg/mol) were purchased from SINOPEG Biotech Co., Ltd. (Xiamen, Fujian, China) and used without purification.

Tetra-PEG-SH and Tetra-PEG-MA were separately dissolved in citrate–phosphate buffer (200 mM, pH 4.6) to obtain polymer solutions. We utilized a Tetra-PEG gel formed from mutually reactive precursors (maleimide-functionalized and thiol-functionalized precursors) with polymer concentration of 40 g/L. This hydrogel shows prolonged stability in vitro and vivo, as it does not include any readily cleavable bonds^22^. The elastic modulus is approximately 5 kPa with a slight temperature dependence^25^. Equivalent volumes of the two solutions were thoroughly combined before application to the rats. The prepared solution was delivered orally to the gastric mucosa via a 12-cm tube device before macroscopic gelation occurred. Here, the gelation time was set at approximately 3–4 min, which enabled ideal gelation just after delivery to the ulcer, taking into account the preparation and operation time. Following delivery, the rats were held in the proper orientation against gravity for 2–3 min to achieve stable gelation.

The results of our preliminary experiments suggested that the optimal time for hydrogel application was 36 h after ulcer induction. Based on this result, Tetra-PEG gel was applied 36 h after ulcer induction in the hydrogel group. In the control group, normal saline was administered using the same method as the hydrogel group. As described in Fig. 1, both groups resumed the same diet as before ulcer induction immediately after application.

### Evaluation of outcomes

After the rats were sacrificed, the stomachs were collected, and the ulcer status was evaluated. To measure the protective effect of hydrogel on ulcers, the following items were evaluated 48 h after gel application: perforation rate, diameter of the ulcer, and ulcer area. As secondary endpoints, the residual rate of hydrogel at the bottom of the ulcer, histological analysis, and adverse events associated with hydrogel were each assessed at 24, 36, and 48 h after gel application. Hematoxylin–eosin staining was conducted on 4-µm sections of post-fixed gastric tissue which was immersed for 24 h in 4% neutral buffered formalin and then immersed for another 24 h in PBS. The gastric tissues were subsequently paraffinized after ethanol dilution series and xylene substitution.

### Statistical analysis

Statistical analysis was conducted using JMP version 11 (SAS Institute Inc., Cary, NC, USA). Categorical data were compared using Fisher’s exact test. The tests were two-sided. *P* < 0.05 was considered statistically significant.

## Supplementary Information

Supplementary Legends.

Supplementary Video 1.

Supplementary Video 2.
