# Supplementary material for: The feasibility of a novel injectable hydrogel for protecting artificial gastrointestinal ulcers after endoscopic resection: an animal pilot study
Source: Sci Rep. 2021 Sep 16;11:18508. doi: 10.1038/s41598-021-97988-9 (PMC8445931; doi:10.1038/s41598-021-97988-9)
Supplement: Supplementary file 1 — Supplementary Legends. [file 41598_2021_97988_MOESM1_ESM.docx]

**Video Clip 1.** Tetra-PEG gel is sprayed using a double-lumen catheter. The instantaneous gelation setting allows for appropriate gelation regardless of the direction of gravity.

**Video Clip 2.** Tetra-PEG gel is sprayed on the gastric mucosa of the pig. The Tetra-PEG gel adhered to the ulcer base, which could not be removed by grasping with forceps.
